# Colon cancer cell invasion is promoted by protein kinase CK2 through increase of endothelin-converting enzyme-1c protein stability

**DOI:** 10.18632/oncotarget.5722

**Published:** 2015-10-16

**Authors:** Ignacio Niechi, Eduardo Silva, Pablo Cabello, Hernan Huerta, Valentina Carrasco, Paulina Villar, Luis Rodrigo Cataldo, Katherine Marcelain, Ricardo Armisen, Manuel Varas-Godoy, Cristina Fernandez, Julio C. Tapia

**Affiliations:** ^1^ Cell Transformation Laboratory, Program of Cellular and Molecular Biology, ICBM, Faculty of Medicine, University of Chile, Santiago, Chile; ^2^ ICBM, Faculty of Medicine, University of Chile, Santiago, Chile; ^3^ Fundacion Ciencia y Vida, Santiago, Chile; ^4^ Department of Anatomopathology, HCUCH, Faculty of Medicine, University of Chile, Santiago, Chile

**Keywords:** CK2, ECE-1, endothelin, colon cancer, metastasis

## Abstract

Endothelin-converting enzyme-1c (ECE-1c) is a membrane metalloprotease involved in endothelin-1 synthesis, which has been shown *in vitro* to have a role in breast, ovary and prostate cancer cell invasion. N-terminal end of ECE-1c displays three putative phosphorylation sites for the protein kinase CK2. We studied whether CK2 phosphorylates N-terminal end of ECE-1c as well as whether this has a role in migration and invasion of colon cancer cells. CK2 phosphorylated the N-terminal end of ECE-1c and this was precluded upon inhibition of CK2. Inhibition also led to diminished protein levels of both endogen ECE-1 or GFP-fused N-terminal end of ECE-1c in 293T embryonic and DLD-1 colon cancer cells, which highlighted the importance of this motif on UPS-dependent ECE-1c degradation. Full-length ECE-1c mutants designed either to mimic or abrogate CK2-phosphorylation displayed increased or decreased migration/invasion of colon cancer cells, respectively. Moreover, ECE-1c overexpression or its silencing with a siRNA led to increased or diminished cell migration/invasion, respectively. Altogether, these data show that CK2-increased ECE-1c protein stability is related to augmented migration and invasion of colon cancer cells, shedding light on a novel mechanism by which CK2 may promote malignant progression of this disease.

## INTRODUCTION

Endothelin-1 (ET-1) is a vasoactive peptide that has been also shown to have a role in cancer [1]. ET-1 is expressed as a large peptide of 212 residues (prepro-ET-1) which is processed intracellularly to big-ET-1 (38–39 residues) [2–3]. Big-ET-1 is secreted into the extracellular media where is processed by the endothelin-converting enzyme-1 (ECE-1) to produce ET-1, the bioactive form of 21 residues [4]. ET-1 may act via their receptors, ET_A_R and/or ET_B_R, in paracrine, endocrine or autocrine ways, activating many signaling pathways involved in cancer progression, including MAPK, NFκB, β-catenin, PI3K/Akt, PKC and Src [1, 5].

Half-life of ET-1 is as short as 1–2 minutes by which either its biological or pathological effects are completely dependent on the conversion of big-ET-1 to ET-1 by the ECE-1 [1]. There are four different isoforms of ECE-1 (a, b, c and d) which have the same transmembrane and extracellular C-terminal catalytic domains, but differ in their cytoplasmic N-terminal ends [6–7]. ECE-1c is mainly expressed in non-tumor and tumor cells but, importantly, is the unique isoform up-regulating invasion in prostate [8] and breast cancer cells [8–10]. This suggests that the role of ECE-1c in cancer cell invasion depends of its cytoplasmic N-terminal end. Interestingly, N-terminal end of ECE-1c displays three putative phosphorylation sites for protein kinase CK2, namely threonine-9, serine-18 and serine-20.

Protein kinase CK2 is an ubiquitous eukaryotic Ser/Thr-kinase that function either as an isolated catalytic subunit (α or α') or in combination with regulatory (β) subunits by forming holoenzymes α_2_β_2_, α′_2_β_2_ or αα′β_2_ [11–12]. The consensus phosphorylation motif for CK2 is S/TXXD/E/^P^S/^P^T/^P^Y (*X* = any, *P* = phosphorylated), which is generally found close to regions rich in acidic residues [13]. This enzyme has more than 300 known substrates [11] and is implicated in regulation of many cellular processes like replication, transcription, translation, proliferation and apoptosis [11, 14–15], many of which are deregulated in cancer [16–17]. CK2 also stimulates the canonical Wnt pathway in colon cancer, where it phosphorylates and stabilizes β-catenin, which promotes expression of key proteins involved in tumor progression, such as survivin, c-myc, COX-2 and endothelin-1 [18–21].

Despite to have three putative CK2-consensus sites, phosphorylation and the effect of this post-translational modification in ECE-1c stability and function have not been studied yet. The aim of this work was to identify a novel target for CK2 and characterize its role in colon cancer malignity. We show here that CK2 enhances protein stability of ECE-1c by phosphorylation of its N-terminal end which promotes migration and invasion of colon cancer cells. In our knowledge, this is the first time where a CK2-dependent regulation of ECE-1c is linked to colon cancer invasion, shedding light on a novel mechanism for this kinase in promoting malignant evolution of the disease.

## RESULTS

### ECE-1c expression is stimulated by CK2 in colon cancer cells

**Figure 1:**
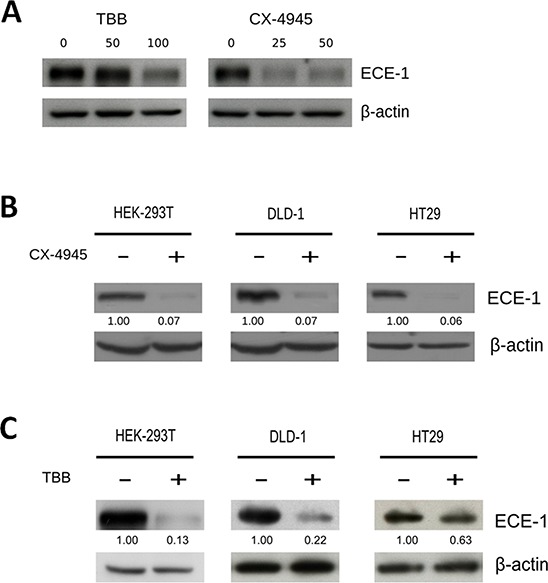
CK2 inhibition decreases ECE-1c protein levels in colon cancer cells **A.** DLD-1 colon cancer cells were incubated in the presence of increasing concentrations of either TBB (0, 50 and 100 μM) or CX-4945 (0, 25 and 50 μM) for 24 h, following detection of ECE-1 protein by western blot with an anti-ECE-1 pan-antibody. DLD-1 and HT29 colon cancer as well as 293T embryonic cells were incubated for 24 h in absence (vehicle) or presence (+) of two specific CK2 inhibitors, 25 μM CX-4945 **B.** and 100 μM TBB **C.** Numbers mean ECE-1 levels normalized to β-actin.

CK2 stimulates the β-catenin-dependent expression of the cancer-related proteins survivin and COX-2, as well as CK2 inhibition decreases their levels and thereby diminishes viability in colon cancer and embryonic cells [20–21]. Here, CK2 inhibition with 4′5′6′7-Tetra-Bromo-2-aza-Benzimidazole (TBB) indeed reduced survivin protein levels in a time- and dose-dependent manner (Supplementary Figure S1A, S1B). As expected, viability of DLD-1colon cancer cells decreased in a dose-dependent fashion by treatment with TBB for 20 h (Supplementary Figure S1C), reaching a similar 60% with 100 μM as published previously [20]. In addition, CK2 inhibition with TBB and also CX-4945 reduced ECE-1 protein levels in a dose-dependent manner in DLD-1 cells (Figure 1A). ECE-1 was also strongly reduced in HT29 colon cancer cells and 293T embryonic cells treated with either 25 μM CX-4945 or 100 μM TBB (Figure 1B, 1C). Since the unique commercially available antibody used here is unable to distinguish ECE-1 isoforms, specific ECE-1c mRNA levels following treatment with TBB were also evaluated. TBB decreased ECE-1c mRNA levels only in 293T cells with no significant effect in colon cancer cells (Supplementary Figure S2A). Moreover, a subtle amplification of a DNA region flanking a putative WRE after immunoprecipitation with either anti-TCF4 or β-catenin antibodies was only observed in 293T cells (Supplementary Figure S2B). Altogether, these results suggest that ECE-1c expression is post-transcriptionally regulated by CK2 in colon cancer cells.

**Figure 2:**
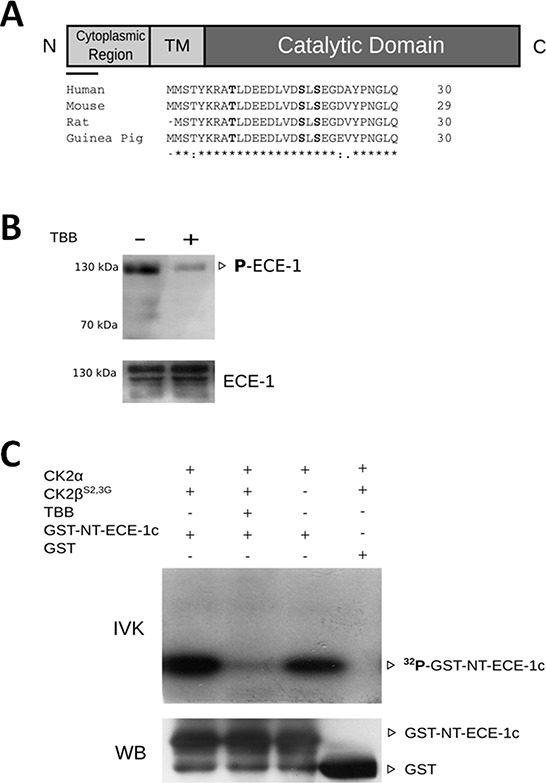
CK2 phosphorylates the N-terminal end of ECE-1c **A.** A schematic representation of ECE-1c, where cytoplasmic N-terminal end, transmembrane (TM) and catalytic domains are depicted. The bar below the N-terminal end corresponds to an alignment of N-terminal end of ECE-1c from several species using ClustalX 2.1. Conserved and putative phosphorylation sites for CK2 are in bold. **B.** In cell phosphorylation performed in DLD-1 cells untreated (−) and treated (+) with 100 μM TBB for 24 h. Protein lysate amounts were adjusted to ensure a similar immunoprecipitation (IP) of ECE-1 in both absence and presence of TBB. Proteins were immunoprecipitated with an anti-ECE-1 pan-antibody, followed of western blot with an anti-phospho-Ser/Thr/Tyr antibody (upper) or an anti-ECE-1 pan-antibody (lower) as a IP control. **C.** Recombinant GST alone (control) or GST-NT-ECE1c proteins were incubated with CK2α and/or CK2β^S2,3G^ in presence of [γ-^32^P]-ATP in an *in vitro* kinase assay (IVK). TBB 100 μM was used to inhibit CK2. ^32^P-labeled proteins were visualized by autoradiography. GST-fused proteins were also detected by western blot (WB) using an anti-GST specific antibody.

### N-terminal end of ECE-1c is phosphorylated by CK2

An *in silico* analysis showed that cytoplasmic N-terminal end of ECE-1c contains three conserved residues fulfilling the phosphorylation consensus for CK2 (Figure 2A). Thus, in order to get a first indication in cell of the ECE-1c phosphorylation and of the involvement of CK2, ECE-1 phosphorylation was evaluated in DLD-1 cells treated or not with TBB. To overcome the decrease in ECE-1 levels observed in TBB-treated cells, lysate amounts were adjusted to ensure a similar immunoprecipitation of this enzyme in both absence and presence of CK2 inhibitor (Figure 2B-bottom). As expected, ECE-1 phosphorylation was found strongly diminished in cells incubated with TBB (Figure 2B). To evaluate the specific phosphorylation of ECE-1c by CK2, cDNA portion of N-terminal end of ECE-1c was cloned and expressed in bacteria fused to GST (ie, GST-NT-ECE-1c). Recombinant CK2 phosphorylated *in vitro* to GST-NT-ECE-1c either as a holoenzyme (ie. CK2α+ CK2β^S2,3G^) or catalytic subunit alone (CK2α). Importantly, phosphorylation of GST-NT-ECE-1c was inhibited with TBB as well as it did not occur with GST alone, which confirmed specificity of phosphorylation by CK2 (Figure 2C). These data show that the N-terminal end of ECE-1c is phosphorylated by CK2.

### N-terminal end of ECE-1c is necessary for its regulation by CK2

**Figure 3:**
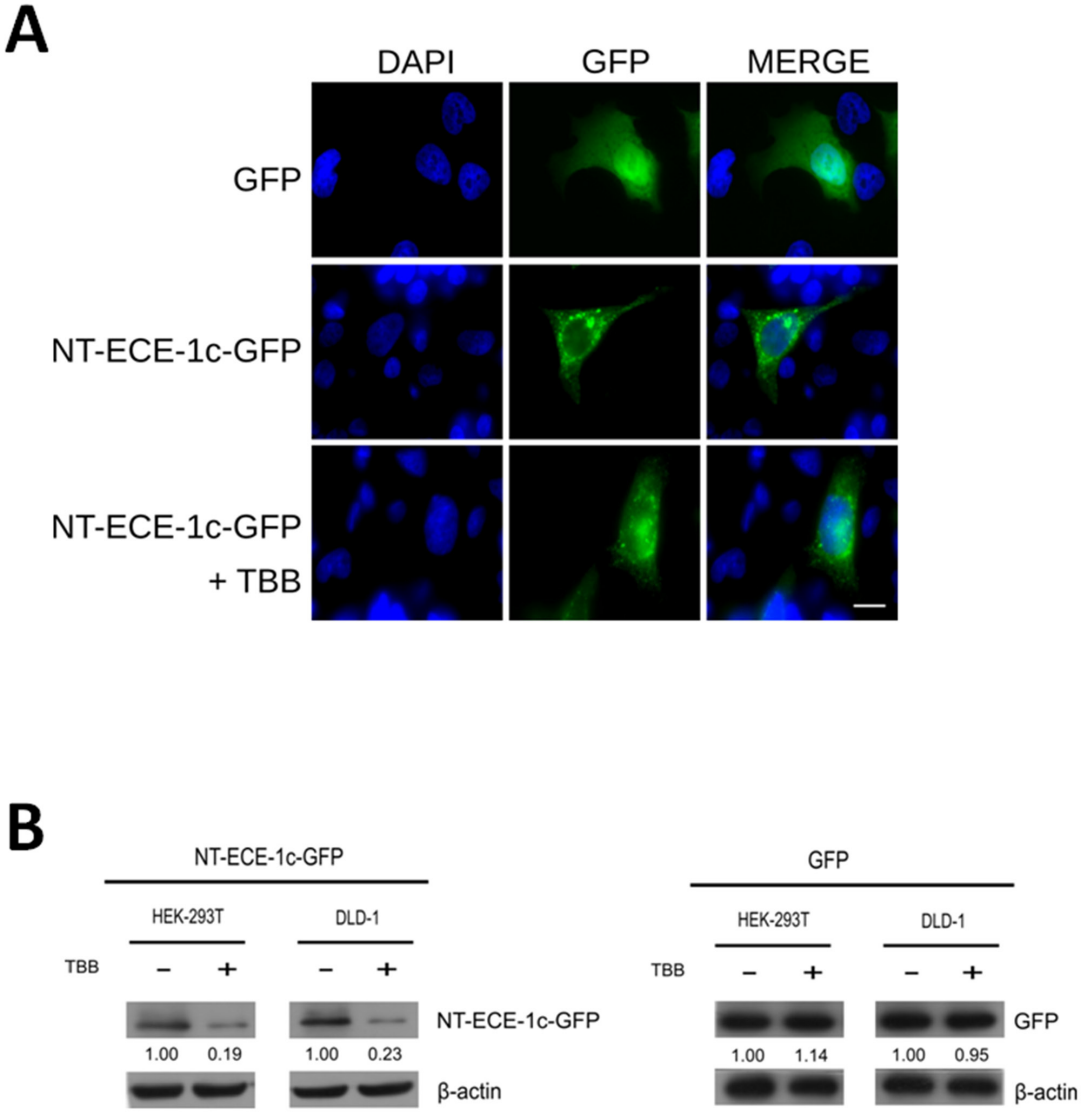
Subcellular localization and protein levels of ECE-1c depend of its N-terminal end **A.** DLD-1 cells expressing GFP alone or NT-ECE-1c-GFP were visualized by confocal microscopy. NT-ECE-1c-GFP expressing cells were treated with 100 μM TBB for 16 h. Bar = 10 μm. **B.** DLD-1 and 293T cells expressing NT-ECE-1c-GFP (left) or GFP alone (right) were untreated (−) and treated (+) with 100 μM TBB for 24 h. Protein levels were detected with a specific anti-GFP antibody. Numbers mean NT-ECE-1c-GFP or GFP protein levels normalized to β-actin as averaged from three independent experiments.

Our findings suggested that phosphorylation by CK2 may protect ECE-1c from proteolytic degradation. To address this, N-terminal end was fused to GFP (ie. NT-ECE-1c-GFP) and expressed in DLD-1 cells. Subcellular localization of NT-ECE-1c-GFP resembled those described for endogenous full-length ECE-1c [22], indicating that the N-terminal end is localizing ECE-1c into its described compartment(s) (Figure 3A). Importantly, treatment with TBB did not significantly alter the localization of NT-ECE-1c-GFP in these cells although its protein levels seemed to decrease. The latter was confirmed when NT-ECE-1c-GFP levels were determined in DLD-1 and 293T cells treated with TBB (Figure 3B), which was very similar to what observed for endogenous full-length ECE-1 (see Figure 1C). These results indicate that the N-terminal end is necessary for the CK2-dependent regulation of ECE-1c protein stability in colon cancer cells.

### ECE-1c proteasome degradation is protected by CK2 phosphorylation in colon cancer cells

**Figure 4:**
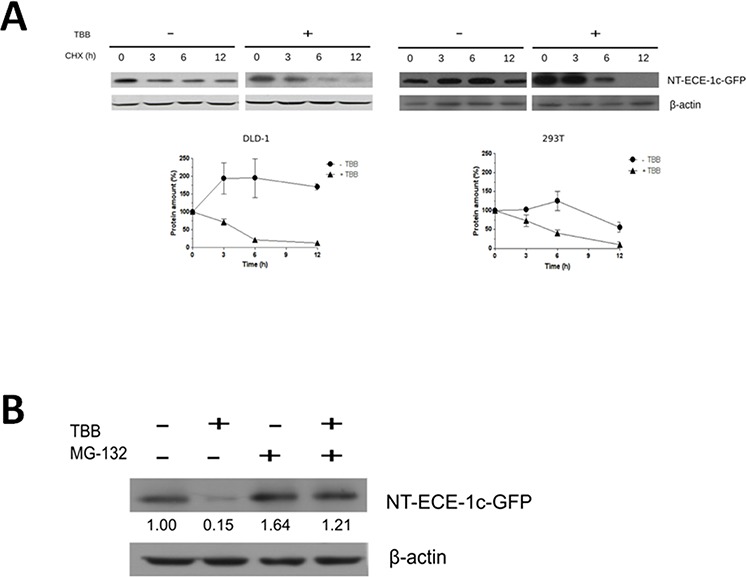
ECE-1c proteasome degradation is promoted by CK2 inhibition **A.** DLD-1 and 293T cells overexpressing NT-ECE-1c-GFP were grown in the absence (−) or presence (+) of 100 μM TBB for 20 h and then treated with 20 μg/mL cycloheximide (CHX) for the indicated times. (Upper) Proteins were detected by western blot using an anti-GFP antibody. (Lower) Pixels from NT-ECE-1c-GFP bands were measured and normalized to β-actin from three independent experiments. **B.** DLD-1 cells expressing NT-ECE-1c-GFP were grown for 20 h in the absence or presence of 100 μM TBB, as well as 10 μM MG-132 to evaluate proteasome degradation. Numbers mean NT-ECE-1c-GFP levels normalized to β-actin as averaged from three independent experiments.

NT-ECE-1c-GFP was expressed in either DLD-1 or 293T cells and its protein stability was evaluated in the presence of TBB and cycloheximide (CHX). CK2 inhibition led to a time-dependent decrease of NT-ECE-1c-GFP levels in both cell lines at a same fashion (Figure 4A). As a control, TBB did not decrease GFP levels in these cells (Supplementary Figure S3A, S3B). Interestingly, NT-ECE-1c-GFP levels in absence of TBB were maintained high in both cell lines but with a most sustained pattern in colon cancer cells. In addition, CK2-increased ECE-1c stability was linked to proteasome degradation in DLD-1 cells, since NT-ECE-1c-GFP levels were restored when the proteasome inhibitor MG-132 was used in the presence of TBB (Figure 4B). Similar results were observed for NT-ECE-1c-GFP in 293T cells (Supplementary Figure S3C), indicating that the N-terminal end of ECE-1c is involved in TBB-induced proteasomal degradation in colon cancer and embryonic cells. Altogether, these results strongly suggest that CK2 phosphorylation at the N-terminal end of ECE-1c may protect it from ubiquitin-proteasome system (UPS) degradation, thereby up-regulating its activity in colon cancer cells.

### Colon cancer cell invasion is improved by CK2-regulation of ECE-1c

**Figure 5:**
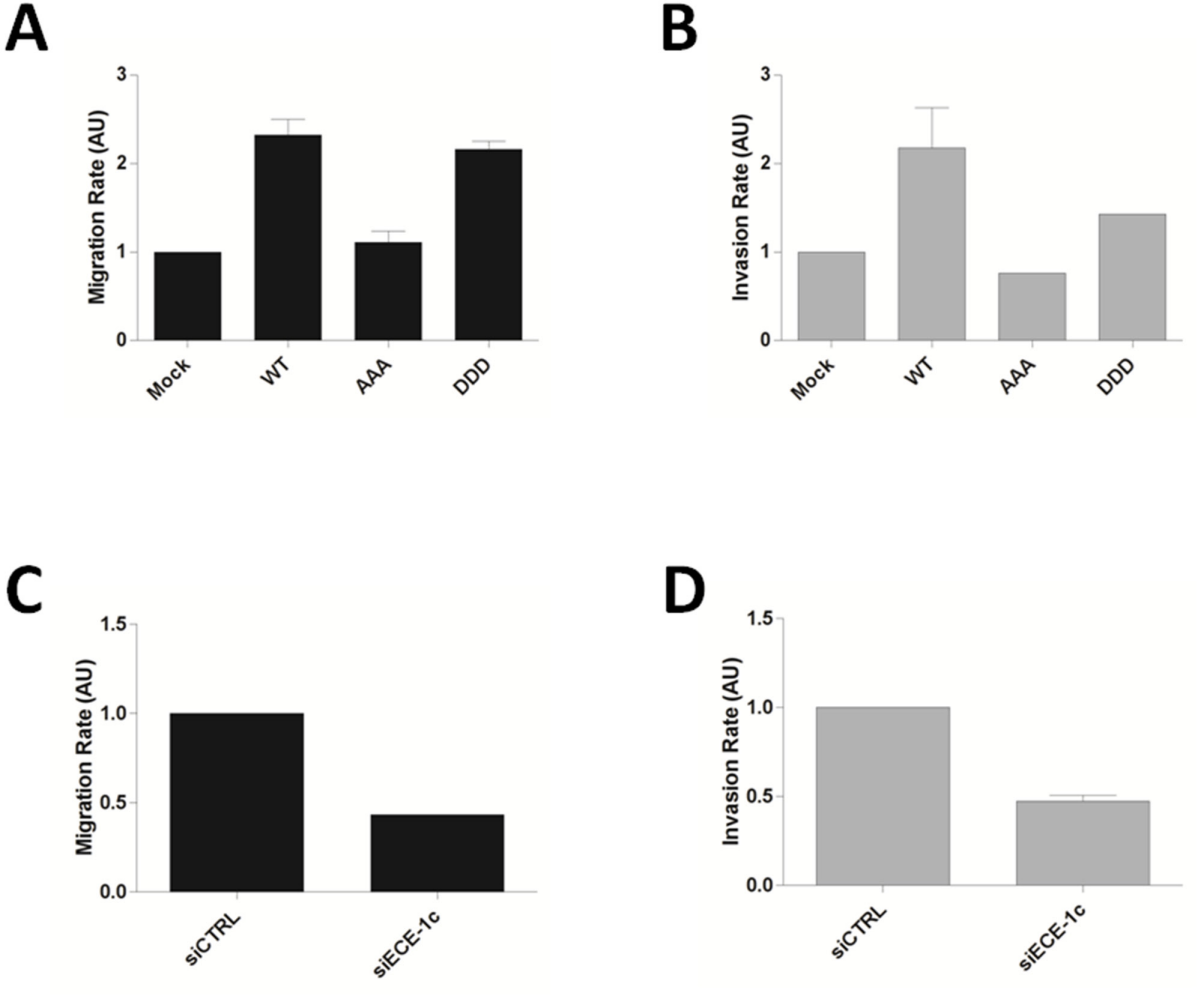
Phosphorylation of N-terminal end of ECE-1c is important for colon cancer cell invasion **A.** DLD-1 cells overexpressing full-length ECE-1c wild-type (WT) and triple-mutant in either T9A/S18A/S20A (AAA) or T9D/S18D/S20D (DDD) were evaluated by a 3D-migration assay. **B.** Cells like in A were evaluated in a matrigel invasion assay. **C.** DLD-1 cells transfected or not with a siRNA for ECE-1c were evaluated in a 3D-migration assay. **D.** Cells like in B were evaluated in a matrigel invasion assay. Data averaged from three independent experiments.

ECE-1c has been shown to improve the *in vitro* invasion of prostate and breast cancer cells [8–10]. Thus, our findings suggested a role of CK2 in colon cancer cell migration and invasion by up-regulating ECE-1c stability. This was evaluated by site-directed mutagenesis of Ser/Thr residues by preparing triple-mutant forms of full-length ECE-1c either mimicking (DDD: T9D/S18D/S20D) or abrogating (AAA: T9A/S18A/S20A) CK2-phosphorylation, as well as by using a siRNA to silence ECE-1c (Supplementary Figure S4). DLD-1 cells viability was not significantly affected for overexpressing the mutants and siRNA (Supplementary Figure S5A, S5B), however, migration significantly augmented when full-length ECE-1c-DDD and WT were overexpressed, while ECE-1c-AAA promoted a lower migration (Figure 5A). As expected, invasion rate correlated with the variations in migration observed for DLD-1 cells overexpressing the ECE-1c mutants (Figure 5B). This probably originated from differences in protein stability of mutants as evaluated in CHO-K1 cells that express negligible ECE-1 levels (Supplementary Figure S5C). In addition, a slightly increased activity of matrix metalloproteinases MMP-2 and −9 was observed by zymography analysis of DLD-1 cells overexpressing ECE-1c (Supplementary Figure S5D). On the other hand, siRNA silencing of endogenous ECE-1c led to diminished both migration and invasion in DLD-1 cells (Figure 5C, 5D). Moreover, inhibition of ECE-1 activity with a generic inhibitor, SM19712, also decreased invasion in DLD-1 and HT29-US colon cancer cells, with no significant effect in viability (Supplementary Figure S6). Taken together, these results demonstrated that ECE-1c is important for invasion of colon cancer cells, which is improved by the CK2-dependent phosphorylation of its N-terminal end and thereby augmented protein stability.

## DISCUSSION

Endothelin-1 (ET-1) has been involved in many cancer-related processes, such as proliferation, angiogenesis, EMT and metastasis, many of these exerted through activation of multiple signaling cascades, including the Wnt/β-catenin pathway and its known targets such as cyclin D1, MMP2 and survivin [1, 20]. In addition, evidence found in the literature sustains that ET-1 is a β-catenin target that participate in an autocrine fashion by up-regulating this signaling pathway, as well as by promoting traits associated with metastasis [19]. Moreover, an *in silico* analysis of the promoter region of ECE-1c performed in our group showed a putative Wnt response element (WRE) −455 bp from the start site +1 (see Supplementary Figure S2B). Thus, given that: (i) a putative WRE exists in the promoter of ECE-1c, (ii) ET-1 is a β-catenin-regulated gene, and (iii) CK2 has been described up-regulating the Wnt/β-catenin pathway [23], altogether this evidence suggested a coordinated expression of both ECE-1c and ET-1 by the CK2/β-catenin axis. Nevertheless, neither TCF nor β-catenin positioning at the putative WRE in the promoter of ECE-1c yielded significant results.

Despite TBB 100 μM was high compared to the *in vitro* IC50, higher concentrations must be used to ensure endogenous inhibition of CK2 in experiments with colon cancer cells, in which a roughly 50% inhibition of the endogenous kinase activity was previously shown for us [20]. Likewise, same concentration of TBB has been also used by other groups to inhibit CK2 in transformed and cancer cells [24–25], as well as to down-regulate the Wnt/β-catenin pathway [21, 26]. However, we observed in this work a decreased ECE-1c promoter-dependent reporter activity but only when assayed in 293T embryonic cells, while GSK3β inhibition with SB291583 (normally used to increase the β-catenin activity) did not lead to a significant increased reporter activity as expected both in embryonic and colon cancer cells (data not shown). Finally, as shown here, similar results with TBB were observed for ECE-1c mRNA levels, albeit CK2 inhibition significantly decreased ECE-1 protein levels in both embryonic and colon cancer cells, suggesting that CK2 promotes ECE-1c expression probably by a post-transcriptional mechanism in colon cancer cells.

The lack of a specific antibody does not allow to ensure that our findings on TBB-dependent decrease of ECE-1 protein levels in colon cancer cells is also occurring for the specific isoform ECE-1c. However, two lines of evidence indicate that this is indeed happening. First, protein levels of both endogenous full-length ECE-1 and N-terminal end fused to GFP (ie. NT-ECE-1c-GFP) were reduced in a similar 78% extent with TBB (compare Figures 1C and 3B). Second, protein stability of overexpressed full-length ECE-1c triple-mutants was significantly different in CHO-K1 cells (Supplementary Figure S5C), which have almost undetectable ECE-1 levels [27].

Activity, cell distribution and trafficking of ECE-1 have been extensively studied by Kuruppu and coworkers [27] whose data indicate that phosphorylation by PKC in the N-terminal end of ECE-1 regulates these processes, which can be altered by conformational changes derived for example of its fusion with GFP. Additionally, phosphorylation of ECE-1 by other protein kinases, including MAPK and CK2, has been suggested albeit not investigated [27]. CK2-dependent phosphorylation has been demonstrated for important proteins involved in cancer [26, 28–29]. Our *in vitro* results showed that CK2 phosphorylates the N-terminal end of ECE-1c by both the catalytic α subunit alone and the holoenzyme, with a subtle increment of phosphorylation when regulatory β subunit was present. Nevertheless, if CK2-dependent phosphorylation occurs in one or more sites of N-terminal end of ECE-1c is yet unknown. Site-directed mutagenesis may be used to identify the precise phosphorylation site(s), but this is time-consuming due to the diverse combinatorial possibilities. Instead, mass spectrometry would be the most suitable approach for defining the precise phosphorylation site(s) as well as the kind(s) of covalent modification(s) targeting ECE-1c for degradation. Despite not having addressed this in our work, our findings are enough convincing to ensure that at least one residue in the N-terminal end of ECE-1c is phosphorylated by CK2.

NT-ECE-1c-GFP displayed a similar subcellular localization in comparison that those described for endogenous ECE-1c [22]. The most plausible explanation for this finding is that only the first 30 residues of N-terminal ECE-1c were sufficient to decrease GFP protein levels when CK2 was inhibited by TBB. This was almost identical to those observed for endogenous ECE-1 in the same cells. Thus, ECE-1c expression was suggested be regulated by a post-translational mechanism of resistance to proteasome degradation, which is promoted by the CK2-dependent phosphorylation at its N-terminal end. In fact, CK2 inhibition with TBB led to a decrease in ECE-1c protein stability in both embryonic and colon cancer cells. Notably, ECE-1c protein levels in presence of only CHX were more strongly stabilized by CK2 in colon cancer than embryonic cells. This was consistent with the elevated CK2 protein and activity levels already detected in different cancer cells, which support the suggested “addiction to CK2” that would occur in cancer cells as proposed elsewhere [30].

There is extensive evidence about the role of ET-1 peptide binding to its receptor ET_A_R in EMT, proliferation, angiogenesis and metastasis [5]. Although limited evidence exists on the role of ECE-1c in invasion and metastasis, which it has been observed mainly expressed in non-tumor and tumor cells, as well as linked to cancer cell progression. Also, overexpressed ECE-1c increases the invasive phenotype in ovarian, breast and prostate cancer cells [8–10, 31]. However, there are no studies addressing the effect of ECE-1c in invasion of colon cancer cells neither if ECE-1c protein stability is increased by phosphorylation. Our results showed that overexpression of a full-length ECE-1c abrogating phosphorylation (ie, AAA) significantly decreased migration/invasion of colon cancer cells, while overexpression of a full-length ECE-1c phosphomimetic for CK2 (ie, DDD) did not significantly increase migration/invasion as compared to the wild-type form. This may be rather a consequence of the higher ECE-1c levels expressed in cancer cells, by which overexpression of a phosphomimetic protein may not significantly influence the migratory capability of these cells. On the other hand, silencing of ECE-1c led to a significant 50% decrease in invasion, which correlated with the observed decrease in mRNA and protein levels. Interestingly, despite to inhibit all isoforms in comparison to the specific silencing of ECE-1c with a siRNA, a similar decrease in invasion was accomplished by using SM19712 in DLD-1 and HT29 colon cancer cells.

Our findings about invasion can be compared with those of ovarian cancer cells. ECE-1 silencing in OVCAR3 and ES2 ovarian lines led to decreased MMP-2 activity, invasion and EMT reversion as shown by both increased E-cadherin and decreased N-cadherin expression, as well as almost 90% decrease in ET-1 secretion [31]. Also, these effects were completely reversed in presence of exogenous ET-1 in ES2 cells but partially in OVCAR3 cells, which suggested that ECE-1 could be exerting its role by continuous production of ET-1 acting on their receptors in ovarian cancer [31]. On the other hand, it has been indicated that ECE-1c increases invasion of PC3 prostate cancer cells in matrigel, but interestingly the addition of purified ET-1 to the media only partially rescued the effect of silencing ECE-1. Thus, an ET-1-independent effect in prostate cancer cell invasion cannot be ruled out for ECE-1c [8]. Whether CK2 promotes colon cancer cell migration and invasion through an ET-1-independent way is an interesting possibility that we cannot rule out with our data. Nevertheless, in our knowledge this is the first time where ECE-1c has been linked to colon cancer invasion as a consequence of its increased protein stability promoted by CK2 phosphorylation at its N-terminal end. In summary, an interesting opportunity may be emerging for ECE-1c as a target for diagnosis and treatment for malignant progression of this disease.

## MATERIALS AND METHODS

### General

Cell medium and antibiotics were purchased from Invitrogen (Paisley, UK). Fetal bovine serum (FBS) was from HyClone (Logan, UT). TBB (4,5,6,7-tetrabromobenzotriazole), cycloheximide (CHX), glutathione-agarose and [γ^32^P]-ATP (6,000 Ci/mmol) were purchased from Sigma-Aldrich (St. Louis, MO). MG-132 was from Calbiochem (Darmstadt, Germany). RIPA buffer and BCA protein kit were from Thermo Scientific (Rockford, IL). Plasmid Midiprep kit and Ni^+2^-NTA-agarose were from Qiagen (Valencia, CA). NitroPure membrane was from Macherey-Nagel (Dϋren, Germany). Lipofectamine 2000 was from Life Technologies (Rockford, IL). Buffers and all other reagents used, but not specified, were from Sigma-Aldrich or the highest grade available.

### Plasmids and siRNA

N-terminal ECE-1c fused to GST was performed using the primers 5′-ggatccatgatgtcgacgtacaagcgggcc-3′ and 5′-gcggccgctccatggagctcaagatggag-3′, which amplify the first 300 nucleotides from human ECE-1c cDNA already cloned into the pcDNA-3 vector. PCR product was subcloned into EcoRI/BamHI sites of pGEX-2T (GE Healthcare, UK). N-terminal variants of ECE-1c fused to GFP were performed using primers 5′-ccggaattccggatgatgtcgacgtacaagcgg-3′ and 5′-cgggatcccgctccatggagctcaagatggag-3′, which amplify the first 300 nucleotides of its sequence. PCR product was subcloned into EcoRI/BamHI sites of pEGFP-N1 (Clontech, CA, USA). Plasmids for expressing recombinant His-CK2α and GST-CK2β in bacteria were described elsewhere [32]. siRNA for ECE-1c, 5′-uucucgauacuacagcugcau-3′, was designed using the online tool at Whitehead Institute and purchased from Ambion (Rockford, IL).

### Cell culture and transfection

Human DLD-1 colon cancer cells were grown in RPMI 1640 medium with 10% FBS and antibiotics (10,000 U/mL penicillin, 10 μg/mL streptomycin; HyClone) at 37°C and 5% CO_2_. Human HT29-ATCC colon cancer and 293T embryonic kidney cells were grown in DMEM high glucose (Invitrogen) with 10% FBS and antibiotics. Lipofectamine 2000 was used for transfections following instructions by the manufacturer.

### Western blot

Blots were probed with the primary antibodies anti-GFP (1:2000) and anti-GST (1:2000) from Sigma; anti-actin (1:2000) from Santa Cruz Biotechnology (Santa Cruz, CA); anti-ECE-1 (1:1000) from Abcam (Cambridge, UK). Detection was performed using secondary anti-goat IgG-HRP (1:2000) or anti-rabbit IgG-HRP (1:2000) from Santa Cruz Biotechnology, and the EZ-ECL chemiluminiscence kit from Biological Industries (Kibbutz Beit, Haemek, Israel).

### In cell phosphorylation

Cell lysates were collected using lysis buffer (1% NP-40, 20 mM Tris pH 7.4, 50 mM NaCl, phosphatase and protease inhibitors). For the immunoprecipitation (IP), 500 μg of proteins were mixed with 2.5 μg of anti-ECE-1 (Abcam) and tubes were incubated with gentle rotation for 3 h at 4°C. Protein A/G agarose (50 μl) was added and samples were incubated for 12 h in the same conditions. IP fractions were obtained by centrifugation at 3500 rpm, 4°C for 2 min. Pellet and soluble fractions were resuspended in loading buffer. Proteins were analyzed by western blot with an anti-phospho-Ser/Thr/Tyr antibody (Abcam) as well as an anti-ECE-1 antibody for IP control.

### Expression and purification of recombinant proteins

Recombinant proteins were purified essentially as described in [32]. Briefly, GST and GST-tagged proteins were expressed in *E. coli* DH5α using 0.3 mM IPTG for 1.5 h at 25°C. His-CK2α was expressed in *E. coli* BL21 (DE3) using 1 mM IPTG for 3 h at 37°C. GST-CK2β^S2,3G^ was expressed in *E. coli* DH5α and purified by using glutathione-agarose. This mutant was shown to interact with and activate CK2α like wild-type CK2β, as well as circumvents the autophosphorylation of the holoenzyme thereby allowing to detect phosphorylation of only added substrates [32]. Purified proteins were separated in 12% SDS-PAGE, identified by western blotting with specific anti-His or GST antibodies, and finally densitometrically quantified by using bovine serum albumin as protein standard. Proteins were aliquoted and stored at −80°C.

### *In vitro* kinase assay

This assay was performed as essentially described in [32]. Briefly, 20 pmol of CK2α were used either alone or combined with an equimolar amount of GST-CK2β^S2,3G^, which reconstitutes the holoenzyme. Assay was carried out in 30 μl reaction volume containing 100 μM [γ-32P]ATP (specific activity 2000–300 cpm pmol^−1^), 10 mM HEPES, pH 7.5, 10 mM MgCl_2_, 0.5 mM DTT, 50 mM NaCl (100 mM for holoenzyme) and 15 pmol GST-NT-ECE1c as substrate. After 30 min incubation at 30°C an aliquot of the mixture was subjected to 12% SDS-PAGE electrophoresis and phosphorylated bands were visualized by autoradiography. The extent of protein phosphorylation was evaluated by densitometric analysis.

### Protein stability

DLD-1 and 293T cells were transfected with NT-ECE-1c-GFP or GFP vectors and grown for 16 h in complete medium. Cells were incubated with 10 μg/ml cycloheximide (CHX) in the presence of 100 μM TBB or vehicle. Cells were harvested at different times for western blotting analysis.

### Confocal microscopy

Following transfection with pEGFP-NT-ECE-1c or pEGFP vectors, cells were tripsinized and grown 24 h on 12 mm coverslips. Cells were treated with TBB or vehicle for 16 h and fixed with 4% p-formaldehyde in 100 mM PIPES buffer pH 6.8 (40 mM KOH, 2 mM EGTA and 2 mM MgCl_2_) for 30 min. Cells were washed three times with PBS and 0.5 mg/ml DAPI was added. Coverslips were mounted on 10% Mowiol, 2.5% 1,4-diazabicyclo [2.2.2] octane. Fluorescent images were obtained by confocal microscopy (Olympus-IX81 DSU Spinning Disk) and merged by using ImageJ.

### 3D-migration and invasion

For 3D-migration assay, bottom side of transwell upper chamber (Corning, Mexico) was coated with 2 μg/ml fibronectin. 50,000 DLD-1 cells were grown with RPMI at 37°C and 5% CO_2_ for 5 h. Migrated cells were fixed and stained with crystal violet/MetOH solution. Non-migrated cells were removed with a cotton-tip. For invasion assay, cells were grown inside a matrigel-coated upper chamber (Biocoat™ Matrigel™, BD) and treated in the same conditions as above but incubated for 22 h. Cells were fixed and stained with 1% toluidine blue/1% borax. In both assays, migrated/invaded cells were counted in seven fields for each experiment.

### Cell proliferation

Colon cancer cell proliferation was determined as published [21]. Briefly, cells were plated in 96-well plate at a density of 1 × 10^4^ cells/well and then treated with the inhibitor at the indicated concentrations for an additional 16–20 h. Alternatively, cells were transfected with indicated plasmids for 24 h and subsequently replated at density indicated above. Cell proliferation was determined by using the MTS® assay (Promega, Madison, WI) according to the manufacturer's instructions.

### ChIP assay

Chromatin immunoprecipitation (ChIP) was performed using a protocol as published elsewhere [33]. Briefly, cells were grown until 60–80% confluence and cross-linked in culture medium at room temperature with 1% formaldehyde solution for 10 min. Cells were lysed and chromatin sonicated on ice with buffer containing protease inhibitors. Four micrograms of either anti-TCF4, β-catenin or IgG (control) antibodies were used for binding, which subsequently were precipitated with protein G-agarose beads. Cross-linking was reversed by incubating at 65°C in an oven for 12 h, treatment with RNase A and proteinase K. Extracted DNA was purified using phenol/chloroform/isoamyl alcohol and precipitated with ethanol. DNA was analyzed by real-time PCR using primers 5′-ccgggtcacactccagt-3′ (fwd) and 5′-ccggcggccccaccgga-3′ (rev).

### Zymography analysis

Cells were grown for 24 h at 60–70% confluence and serum starved for 16 h. Supernatants were collected and concentrated by SpeedVac. Samples were incubated in non-reducing buffer (0.4 M Tris HCl pH 6.8, 5% SDS, 20% glycerol, 0.03% bromophenol blue) for 30 min. Proteins (30 μg) were loaded on 8% SDS-PAGE co-polymerized with 1 mg/ml gelatin. After electrophoresis, gel was incubated with 2.5% Triton X-100 to remove SDS. Subsequently, gel was incubated for 24 h at 37°C in gelatinase activity buffer (150 mM Tris HCl pH 7.5, 150 mM NaCl, 5 mM CaCl2, 0.02% NaN_3_). Finally, gel was stained with 2.5% Coomassie Blue to show gelatin degradation by metalloproteinases MMP-2 and -9, which is observed as a white band.

### Statistical analysis

All results were obtained of at least three independent experiments. When required, data were compared using the Dunnett's or Student's methods after ANOVA. A value for *P* < 0.05 was considered significant.

## SUPPLEMENTARY FIGURES


