# Supplementary material for: Colon cancer cell invasion is promoted by protein kinase CK2 through increase of endothelin-converting enzyme-1c protein stability
Source: Oncotarget. 2015 Oct 16;6(40):42749–60. doi: 10.18632/oncotarget.5722 (PMC4767467; doi:10.18632/oncotarget.5722)
Supplement: Supplementary file 1 [file oncotarget-06-42749-s001.pdf]

SUPPLEMENTARY FIGURES

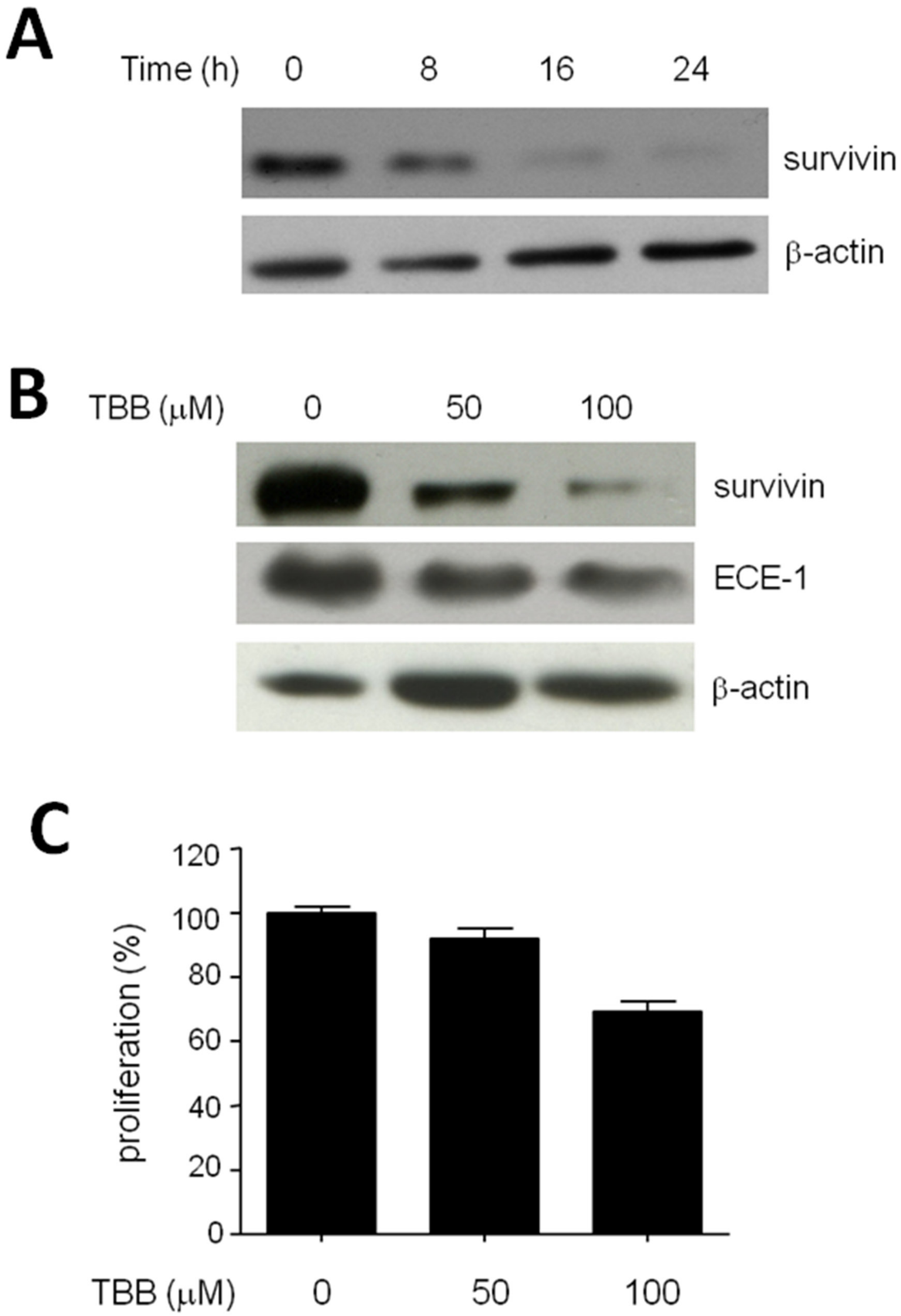

Supplementary Figure S1: CK2 inhibition decreases viability in colon cancer cells. A. DLD-1 colon cancer cells were incubated in presence of the specific CK2 inhibitor TBB 100 μM for the indicated times, following detection of survivin protein by western blot with a specific antibody. **B.** DLD-1 cells were incubated for 20 h in presence of increasing concentrations of TBB, following detection of survivin and ECE-1 proteins by western blot. **C.** DLD-1 cells were incubated as in B and proliferation was determined by the MTS® assay.

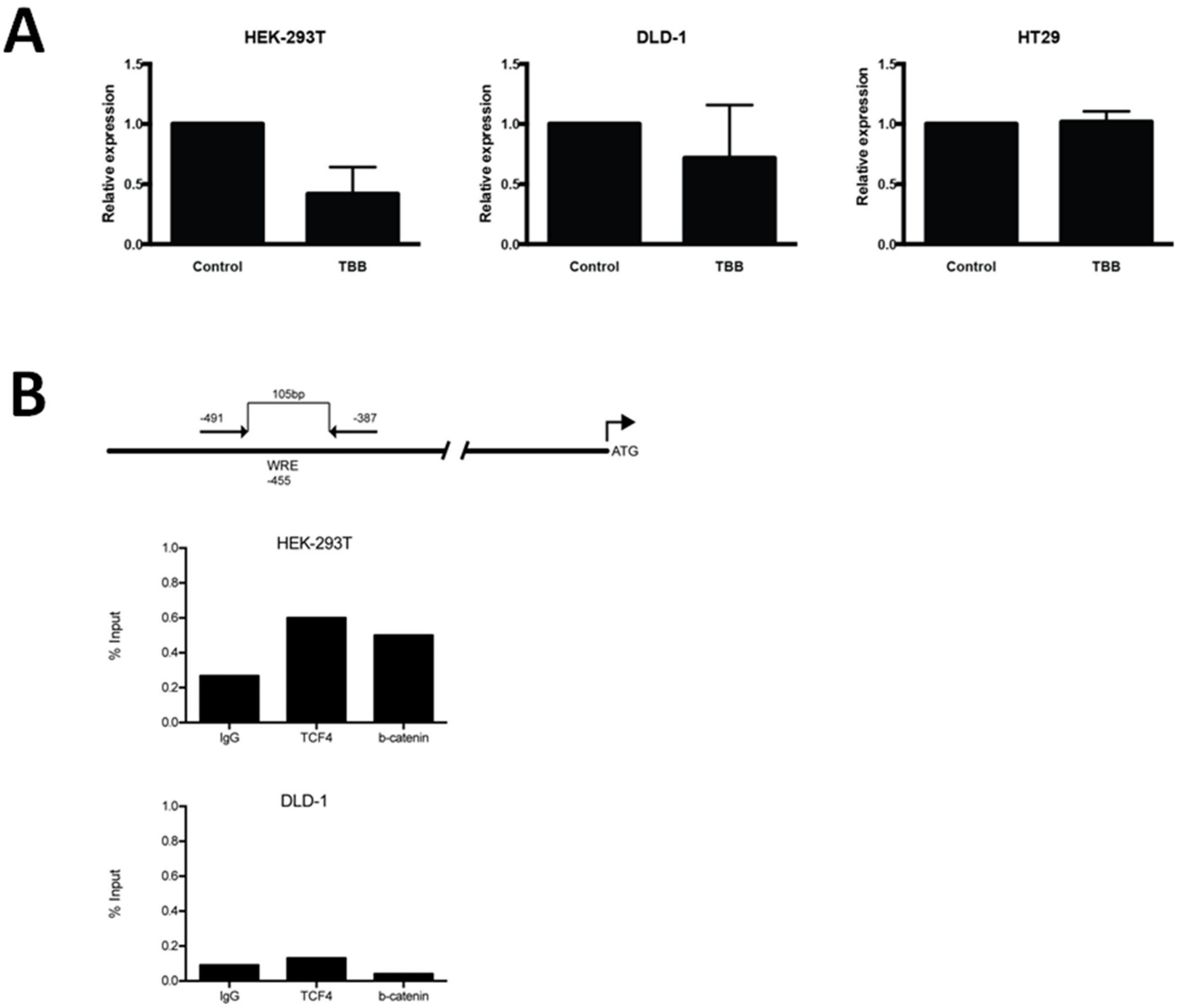

Supplementary Figure S2: ECE-1c is post-transcriptionaly regulated by CK2 in colon cancer cells. A. DLD-1 and HT29 colon cancer as well as 293T embryonic cells were incubated for 24 h in absence (control, vehicle) or presence of 100 µM TBB. ECE-1c mRNA levels were detected by using RT-qPCR, where 18S rRNA was used to normalize relative levels. **B.** TCF-4 and β-catenin binding to the ECE-1c promoter was analyzed by ChIP in DLD-1 and 293T cells. Isolated chromatin was immunoprecipitated using anti-TCF4 or anti-β-catenin antibodies. (Upper) ECE-1c promoter region containing a putative Wnt response element (WRE) and the primers used for qPCR analysis. (Lower) Enrichment of chromatin was analyzed by qPCR using primers flanking depicted region.

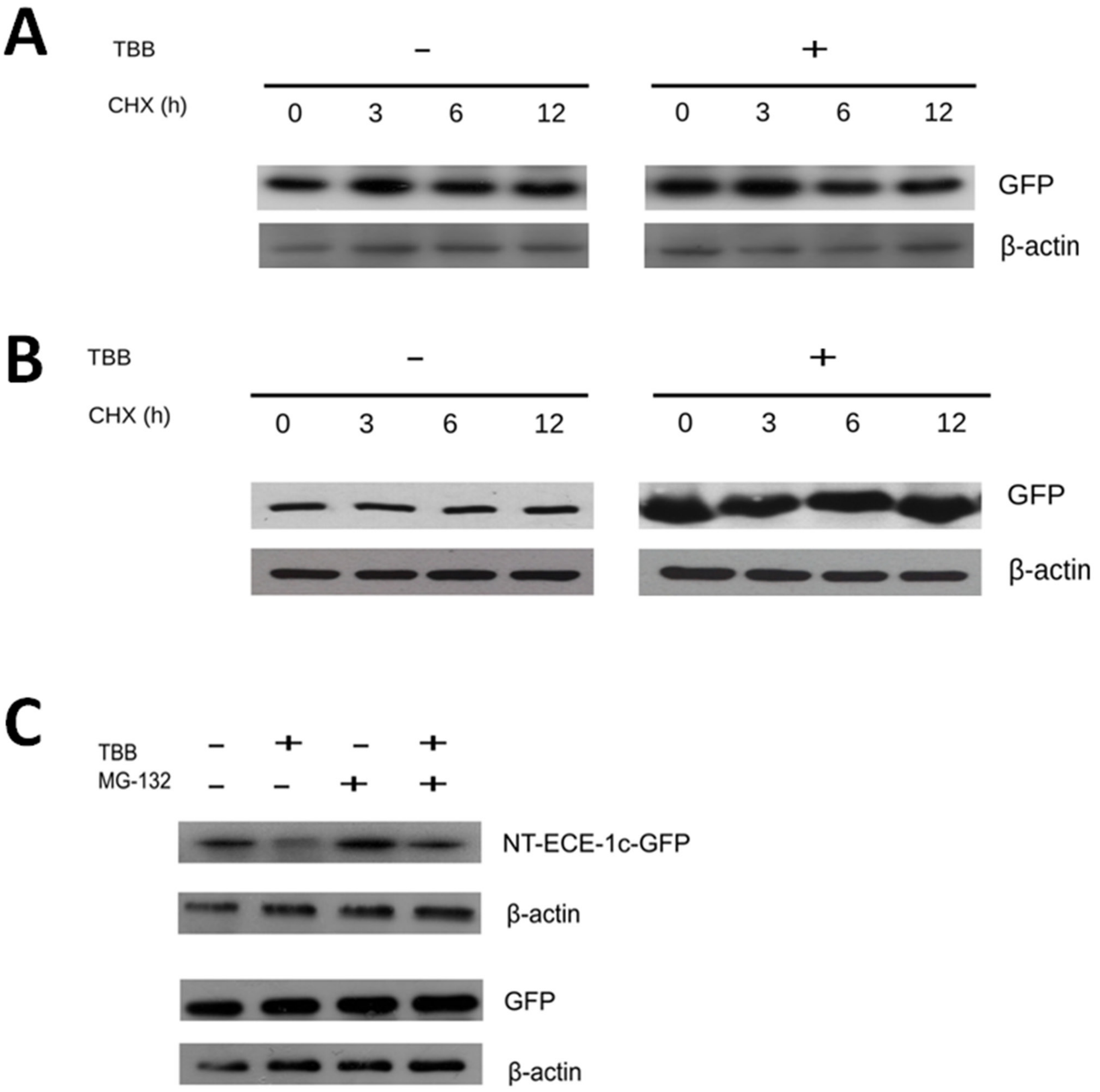

Supplementary Figure S3: N-terminal end of ECE-1c is important for CK2-promoted proteasome degradation in DLD-1 and 293T cells. A. DLD-1 cells expressing GFP were treated with 20 µg/ml CHX in the absence (−) or presence (+) of 100 µM TBB for 20 h. Proteins were detected at indicated times by western blot using a GFP-specific antibody. **B.** 293T cells expressing GFP were evaluated in the same conditions as in A. **C.** 293T cells expressing NT-ECE-1c-GFP (Upper) or GFP alone (Lower) were grown for 20 h in the absence (−) or presence (+) of either 100 mM TBB or 10 mM MG-132 as indicated. Lysates from cells were analyzed by western blot using b-actin as control.

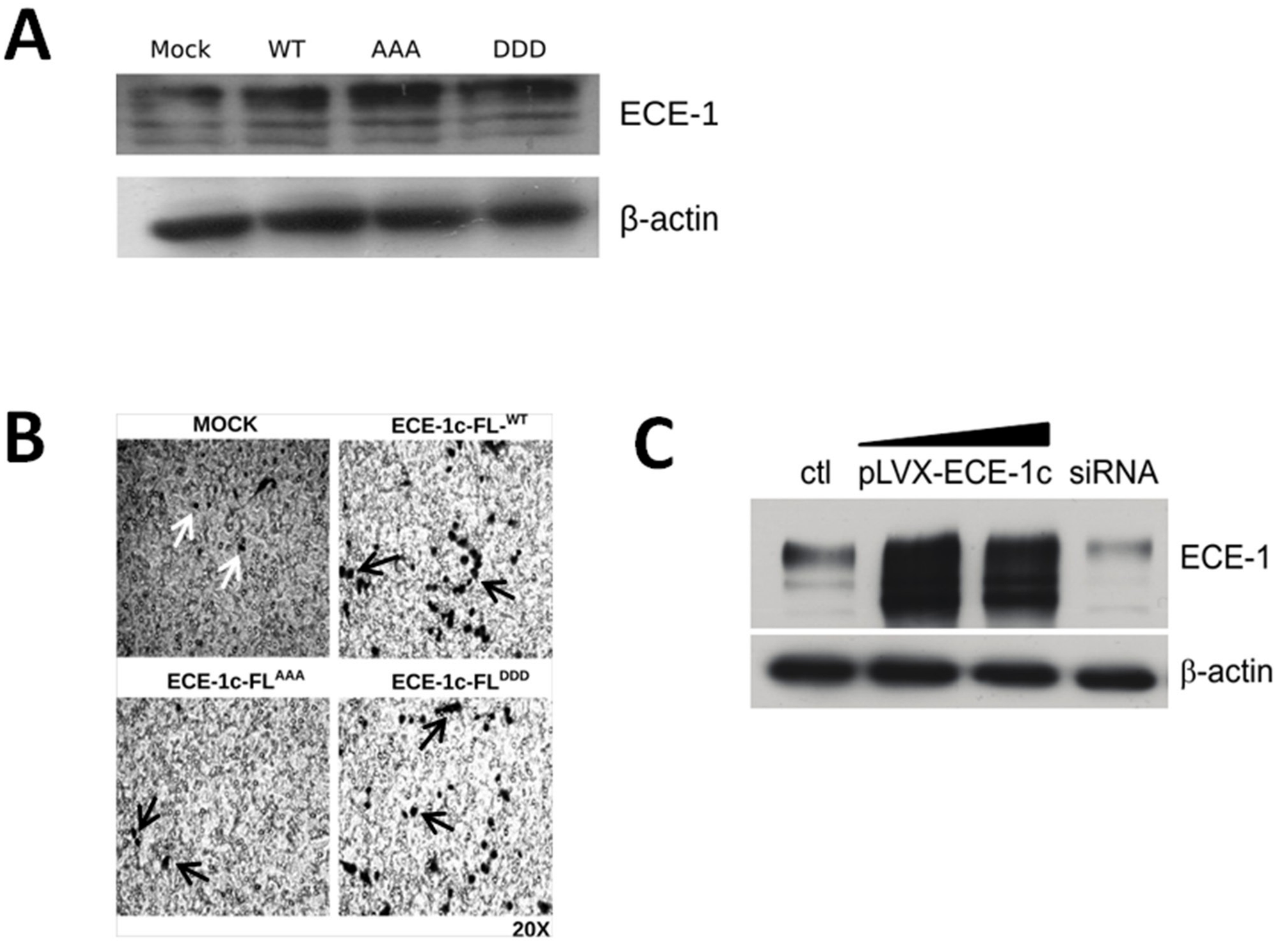

Supplementary Figure S4: Ectopic expression of full-length ECE-1c variants and mRNA silencing in colon cancer cells. A. Full-length ECE-1c mutated in either T9D/S18D/S20D (DDD), T9A/S18A/S20A (AAA) or wild-type (WT) were expressed in DLD-1 cells. ECE-1 protein level was evaluated by western blot with an anti-ECE-1 antibody. **B.** Representative images of cells in a 3D-migration assay for all conditions in 5A. Arrows indicate migrated cells stained with crystal violet/MetOH solution. **C.** Lysates from DLD-1 cells transfected with increasing amounts of plasmids encoding either full-length ECE-1c WT or a siRNA were analyzed by western blot using indicated antibodies.

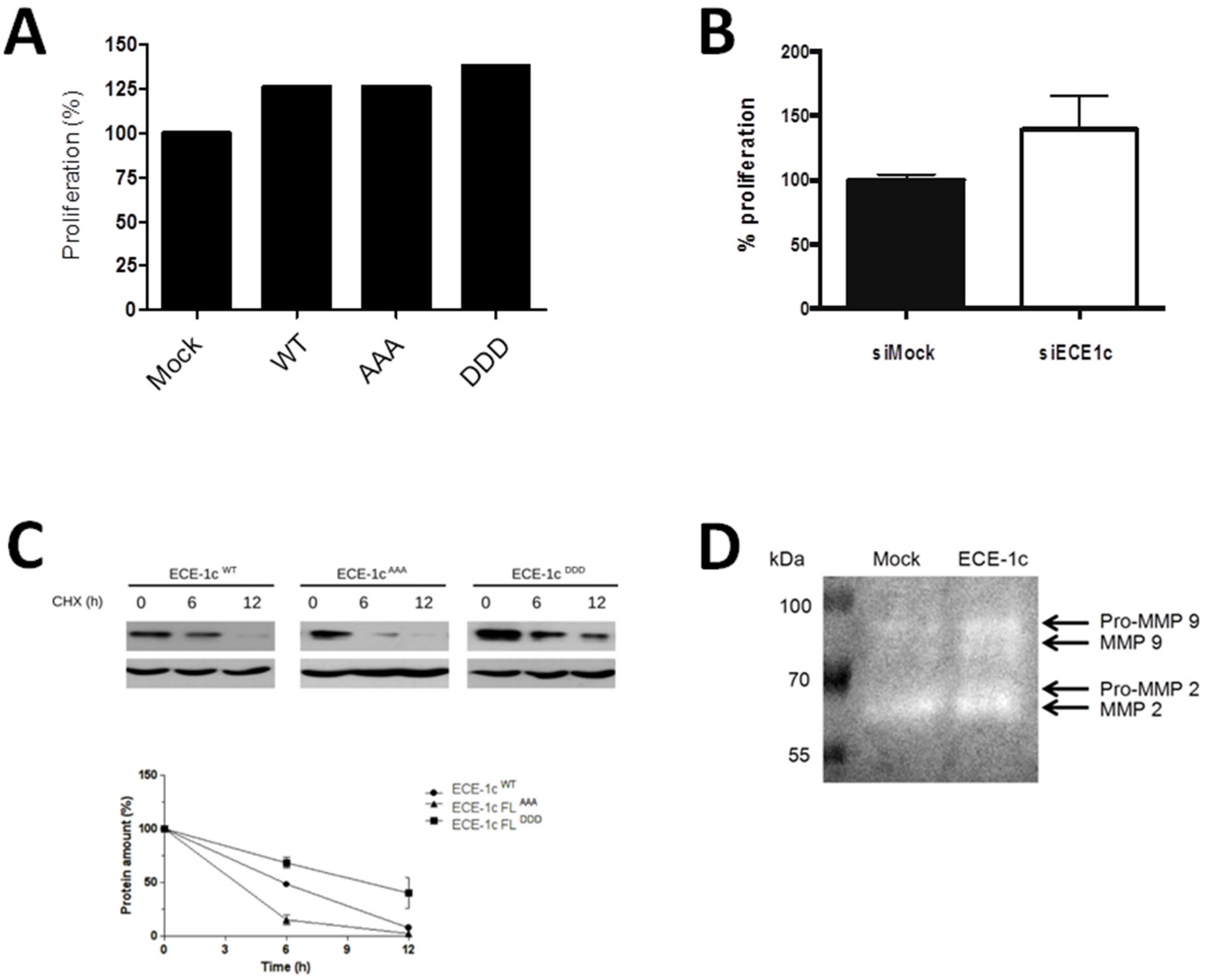

Supplementary Figure S5: Effect of ECE-1c wild-type, mutants and siRNA in proliferation and protein stability in colon cancer cells. A. Proliferation was evaluated by MTS assay in DLD-1 cells transfected with plasmids encoding full-length ECE-1c WT and N-terminal end mutants. **B.** Proliferation was evaluated by MTS assay of DLD-1 cells transfected with a specific siRNA for ECE-1c. **C.** CHO-K1 cells expressing ECE-1c wild-type and mutants were treated with 20 µg/ml CHX at different times. Proteins were detected at indicated times by western blot using an anti-ECE-1 antibody. **D.** Zymographic analysis of DLD-1 cells transfected with empty plasmid (mock) or a vector encoding full-length ECE-1c WT. Supernatants from growing cells were separated on SDS-PAGE co-polymerized with gelatin. After removing SDS, gel was incubated in activation buffer to show gelatin degradation by MMPs through comassie blue staining.

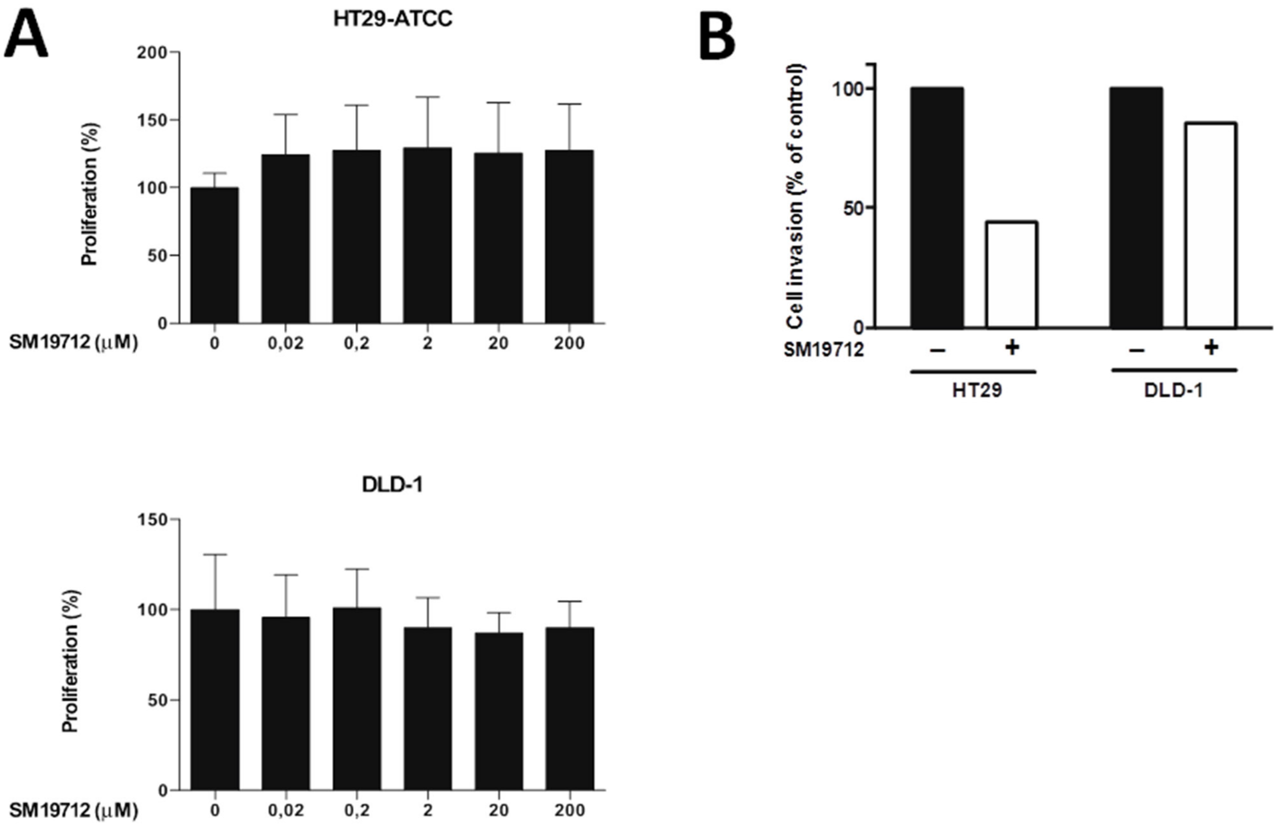

Supplementary Figure S6: Effect of ECE-1 pharmacological inhibition in proliferation and invasion of colon cancer cells. A. Proliferation was measured by MTS assay in DLD-1 and HT29 cells incubated with increasing concentrations of ECE-1 inhibitor, SM19712, for 21 h. Data averaged from three independent experiments. **B.** Invasion was evaluated by matrigel assay in DLD-1 and HT29 colon cancer cells treated with 100 nM SM19712 for 21 h.
